# Bacterial Glycocalyx Integrity Impacts Tolerance of *Myxococcus xanthus* to Antibiotics and Oxidative-Stress Agents

**DOI:** 10.3390/biom12040571

**Published:** 2022-04-12

**Authors:** Fares Saïdi, Razieh Bitazar, Nicholas Y. Bradette, Salim T. Islam

**Affiliations:** 1 Centre Armand-Frappier Santé Biotechnologie, Institut National de la Recherche Scientifique (INRS), Université du Québec, Institut Pasteur International Network, Laval, QC H7V 1B7, Canada; fares.saidi@inrs.ca (F.S.); razieh.bitazar@inrs.ca (R.B.); nicholasbradette@gmail.com (N.Y.B.); 2 PROTEO, the Quebec Network for Research on Protein Function, Engineering, and Applications, Université Laval, Québec, QC G1V 0A6, Canada

**Keywords:** biofilms, extracellular matrix, antibiotic resistance, antibiotic tolerance, reactive oxygen species (ROS), Wzx/Wzy-dependent pathway, multicellularity, oxidative stress, exopolysaccharide, biosurfactant polysaccharide (BPS)

## Abstract

The presence of an exopolysaccharide (EPS) layer surrounding bacterial cells, termed a “glycocalyx”, confers protection against toxic molecules. However, the effect of glycocalyx integrity on the tolerance to such agents is poorly understood. Using a modified disc-diffusion assay, we tested the susceptibility to a panel of antibiotics and oxidative stress-inducing compounds of various mutant strains of the social predatory Gram-negative soil bacterium *Myxococcus xanthus*; the selected mutants were those that manifest different physical states of their respective EPS glycocalyces. While the overall presence of an EPS layer was indeed beneficial for tolerance, the integrity of this layer was also found to affect the susceptibility of the bacterium to killing; however, this finding was not universal, and instead was dependent on the specific compound tested. Thus, the integrity of the cell-surface EPS glycocalyx plays an important role in the tolerance of *M. xanthus* to harmful compounds.

## 1. Introduction

Secreted high-molecular-weight (HMW) polysaccharides play integral roles in various stages of growth and development for diverse bacteria. Surface-associated arrangements of such polymers can form a localized matrix around cells termed a “glycocalyx”, a structure implicated in the formation of biofilm aggregates that promote community endurance during detrimental environmental stimuli [1].

An important protective effect of the physical barrier afforded by a biofilm lifestyle extends to the tolerance of antibiotics and other stress-inducing compounds. In some instances, existence within a biofilm matrix composed of HMW polysaccharides can reduce bacterial susceptibility to these compounds by a factor of 100 to 1000 [2]. Antibiotic tolerance differs in a fundamental way from antibiotic resistance. Tolerance stems from a non-specific increase in resilience to treatment with a given compound resulting from an altered phenotypic state (such as existence in a biofilm). Conversely, resistance implicates dedicated mechanisms (e.g., inactivating enzymes, efflux pumps, etc.) that lower the effectiveness of a compound at a given concentration [2]. While antibiotic tolerance can lead to antibiotic resistance, the former topic is far less studied than the latter topic.

The social Gram-negative soil bacterium *Myxococcus xanthus* follows a complex multicellular lifecycle [3] in which it can predate other bacteria in a contact-dependent manner [4] and exhibit saprophytism through use of the degradation products. Single-cell gliding [5,6] and collective group motility [7,8], mediated by the Agl-Glt and type IV pilus (T4P) machineries (respectively) are important for the physiology of the bacterium. Upon exposure to low-nutrient conditions, cells in a swarm biofilm enter a developmental program in which they undergo differentiation to form fruiting-body structures containing myxospores [3].

**Figure 1:**
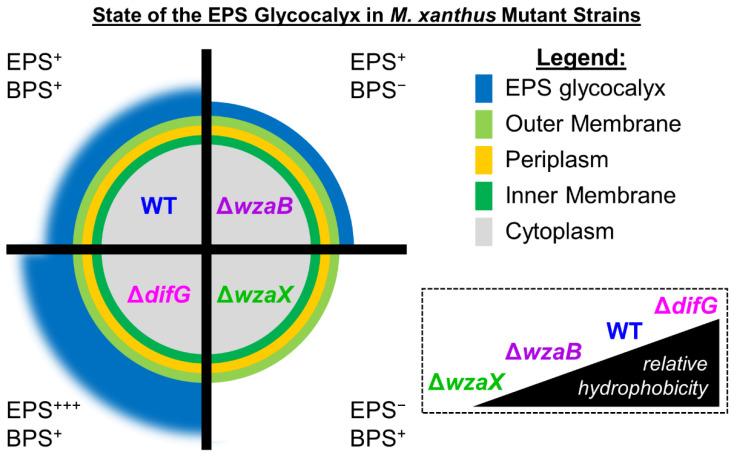
State of the EPS glycocalyx in *M. xanthus* mutant strains. The cell is depicted in an end-on view, looking down the long axis of the rod-shaped cell. WT cells producing EPS and BPS have a disrupted cell-surface EPS glycocalyx. Cells deficient for Class 3 OPX protein WzaB (BPS^−^) still produce WT amounts of cell-surface EPS, but this EPS is present in a more contiguous, non-functionally-activated state. Cells deficient for DifG over-produce cell-surface EPS. Cells lacking Class 3 OPX protein WzaX still produce BPS, but are completely deficient for cell-surface EPS. *Inset:* relative cell-surface hydrophobicity of various mutant strains, as previously determined via assays measuring cell adhesion to hexadecane [7,8].

Three HMW secreted polysaccharides are known to play important roles in this intricate lifecycle [9]. For developmentally-engaged cells, major spore coat (MASC) polymer is produced, which surrounds myxospores in a protective layer. For vegetative cells, exopolysaccharide (EPS) is secreted; this polymer forms a surface-associated glycocalyx that coats the entire cell body [8]. The non-capsular nature of this polymer also results in EPS being the principal component of biofilm matrices in this bacterium [10,11]. Finally, a novel biosurfactant polysaccharide (BPS) is produced and secreted to the local extracellular environment [7], where it destabilizes the integrity of the EPS glycocalyx [8] (Figure 1). A BPS-destabilized EPS glycocalyx is an important mediator of *M. xanthus* physiology at the single-cell level as it impacts relative hydrophobicity of the cell surface, gliding speed, and gliding reversal frequency, as well as the production, positioning, and stability of T4P structures [8]. At the community level, different spatiospecific patterns of EPS and BPS production influence the internal structure of swarm biofilms, with the interplay between EPS and BPS also impacting the responsiveness of the swarm community to mechanical changes in the substratum [7,8].

EPS, BPS, and MASC are produced by separate Wzx/Wzy-dependent pathways [7,12,13] with individual components given the suffixes X (exopolysaccharide), B (biosurfactant), or S (spore coat). Therein, a Wzx flippase translocates undecaprenyl pyrophosphate (UndPP)-linked sugar repeats between the cytoplasmic and periplasmic leaflets of the inner membrane (IM) [14,15,16]. Repeats are then polymerized by Wzy at the periplasmic leaflet of the IM [17,18] to lengths regulated by Wzc polysaccharide co-polymerase (PCP) proteins [19,20]. Polymers are then proposed to exit a channel formed by PCP proteins and interact with outer-membrane (OM) polysaccharide export (OPX) proteins in the periplasm [21]. For the canonical *Escherichia coli* Group 1 capsule OPX protein Wza (Wza*_Ec_*), multimers form an enclosed channel to continue transport of polymer across the periplasm and OM, the latter by way of an α-helical integral-OM pore [22,23]. While widespread, we recently reported Wza*_Ec_*-like architecture (Class 1) to be the second-most common among three defined OPX-protein classes in all Gram-negative and Gram-positive bacteria. Instead, the *M. xanthus* WzaX, WzaB, and WzaS OPX proteins were all identified as prototypic Class 3 OPX proteins, the most predominant in all bacterial systems; proteins of this OPX class lack a C-terminal OM-spanning α-helical domain [24]. Instead, genes for WzaX/B/S were all genomically paired with adjacent genes encoding WzpX/B/S integral-OM β-barrel porins for translocation of EPS/BPS/MASC (respectively) across the OM [7,24]. Similar β-barrel porins were found to be encoded near OPX genes of all three structural classes in a range of bacteria, pointing to a widespread secretion paradigm in pathways involving OPX proteins [24].

Pointing to the importance of the polymer for the bacterium, EPS biosynthesis in *M. xanthus* is subject to complex regulation. Bacterial tyrosine kinases in each *M. xanthus* Wzx/Wzy-dependent pathway are essential for production of the respective polymer [7], and are known (or proposed) to be dephosphorylated by a bacterial tyrosine phosphatase [8,25]. This phosphorylation dynamic has recently been shown to play an important role in HMW polysaccharide assembly and secretion [21]. In addition, the Dif pathway plays a major role in EPS regulation (reviewed in [9]). While various factors either positively or negatively regulate EPS production, the latter is significantly impacted in the absence of the CheC-like phosphatase DifG, resulting in substantially higher levels of EPS production in a Δ*difG* mutant strain [8]. Despite the importance of EPS, the effect of its modulation on *M. xanthus* tolerance to harmful compounds is poorly understood.

Herein, we adapted a classic disc-diffusion assay to probe differences in susceptibility to various antibiotics and oxidative stress-inducing compounds for *M. xanthus* mutant strains with different cell-surface glycocalyx properties (Figure 1). In so doing, we reveal that not just the overall presence/absence of an EPS glycocalyx, but the integrity of the glycocalyx layer itself, are important mediators of tolerance to toxic compounds.

## 2. Materials and Methods

### 2.1. Bacterial Cell Culture

Genomic details [26] of the wild-type *M. xanthus* DZ2 and isogenic polysaccharide-mutant strains analyzed herein can be found in Table 1. Strains were streaked from sporulated freezer stocks on CYE [27] agar plates (1% *w*/*v* casitone, 0.5% *w*/*v* yeast extract, 10 mM MOPS ([pH 7.5], 4 mM MgSO_4_, 1.5% *w*/*v* agar [BD Difco]) and grown at 32 °C. CYE liquid cultures were grown at 32 °C on a rotary shaker (220 rpm).

### 2.2. Disc-Diffusion Assay

Liquid CYE cultures (20 mL) were grown overnight (32 °C, shaking at 220 rpm). The following day, the OD_600_ of each culture was determined using a disposable acrylic cuvette in a spectrophotometer, using 100 µL of culture mixed with 900 µL of TPM buffer (10 mM Tris-HCl [pH 7.6], 8 mM MgSO_4_, 1 mM KH_2_PO_4_); continued aspiration in these conditions allowed for homogenization of the resuspension through the dissociation of cell aggregates. Based on the obtained density value, sufficient volume of overnight culture was removed and sedimented via centrifuge (10,000× *g*, 5 min) so that concentrated resuspension in 1 mL would yield an OD_600_ of 15. Culture supernatant was thus removed, followed by resuspension in 1 mL CYE. To prepare top agar, 800 µL of concentrated cell resuspension was mixed with 600 µL of molten CYE 1.5% agar and 1,600 µL of CYE liquid medium; this mixture was poured on top of a matrix of pre-solidified CYE 1.5% agar (20 mL) in a round disposable Petri dish (92 mm × 16 mm). Plates were left uncovered in a biosafety cabinet for 30 min to allow for solidification of the top-agar overlay. Using sterilized tweezers, 4 or 5 autoclaved Whatman Antibiotic Assay Discs (Fisher Scientific, 2017-006, Ottawa, Canada) were placed at equidistant positions from each other on the agar surface. Plates were left to sit another 15 min to allow for discs to firmly associate with the agar surface. Next, 10 µL of a given compound at a given concentration was dispensed atop each disc, followed by a 15 min waiting period to ensure absorption of the added volume by the disc. Plates were subsequently sealed with Parafilm, and incubated face-down at 32 °C for 48 h. Finally, to image clearance zones, plates were scanned on a GelDoc (Syngene Chemi Genius 2 [Genesnap, Zoom 1.2, Focus 151, Lens aperture 1.2], Frederick, MD 21704, USA). For each biological replicate, two radial clearance distances were measured per disc with ImageJ, with the mean representing the clearance distance. All tests were carried out in biological triplicate. Data displayed normal distributions (Shapiro-Wilk test). Two-way ANOVA with Dunnett’s multiple comparisons test (α < 0.05) was used to compare means of mutants against WT at each compound concentration tested. GraphPad was used for all statistical analyses.

All compounds were dissolved in autoclaved ddH_2_O, then sterilized through a 0.2 µm syringe filter. Antibiotics were prepared at the following stock concentrations (then diluted): ampicillin, 100 mg/mL (Bio Basic, AB0064, Markham, Canada); chloramphenicol, 25 mg/mL (Fisher Bioreagents, Bp 904-100, Ottawa, Canada); ciprofloxacin 100 mg/mL (MP Bio, 199020, Solon, OH 44139, USA); polymyxin B sulfate, 50 mg/mL (MilliporeSigma, 5291, Oakville, Canada); vancomycin hydrochloride hydrate, 10 mg/mL (MilliporeSigma, 861987, Oakville, Canada). Stock solutions of oxidative stress-inducing molecules were prepared as follows: ammonium persulfate, 1 M (Fisher Scientific, BP179-100, Ottawa, Canada); hydrogen peroxide, 50% *w*/*w* (MilliporeSigma, 516813, Oakville, Canada); methyl viologen hydrate (i.e., Paraquat), 1 M (Acros Organics, 227320010, Morris Plains, NJ 07950, USA).

## 3. Results

**Figure A1:**
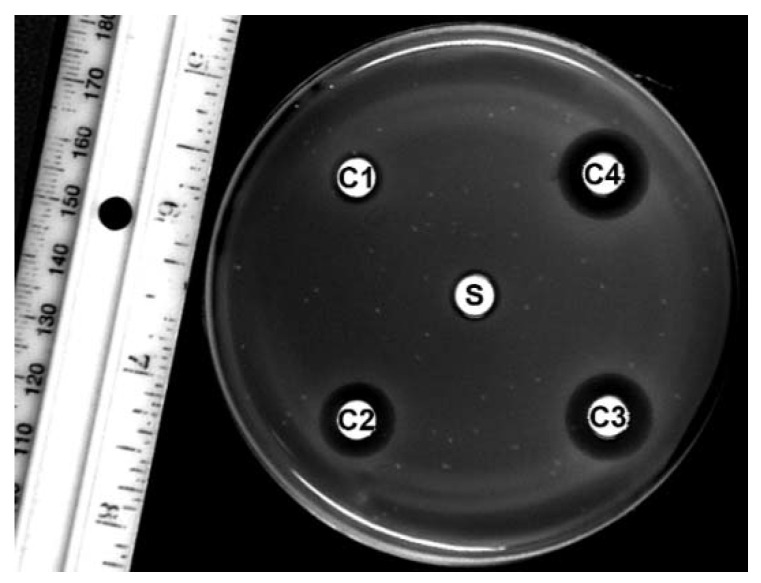
Example image from the disc-diffusion assay. To each filter disc on the *M. xanthus*- inoculated top-agar surface, equivalent volumes of pure solvent (S), and increasing concentrations of a particular compound dissolved in the same solvent (C1, C2, C3, C4), were added, followed by clearance zone radius measurement after incubation. (This image: WT, 200/400/600/800 mM APS).

To compare differences in antibiotic susceptibility between bacterial strains, minimum inhibitory concentration (MIC) drug-titration assays are often carried out with shaking incubation of small culture volumes arrayed on multi-well plates. Alternatively, colony-forming units (based on the assumption that a single cell gives rise to a new colony) are determined post-treatment with compounds at different concentrations. However, given the propensity of *M. xanthus* to form aggregates in liquid culture and biofilms on culture vessel walls, as well as its proficiency at spreading on agar surfaces, we avoided such approaches. Instead, we adapted the widely-used disc-diffusion assay for use with this bacterium in which zones of clearance around filter discs impregnated with various compounds can be quantified and compared (Figure A1). Of note, differences in surface morphology between WT and polysaccharide-secretion mutants can be observed in both liquid-grown and swarm biofilm-grown cells [8]. Therefore, to preclude any effects on clearance zones from differences in inter-strain surface motility, rather than spreading strain inoculum on the surface of an agar plate, we first mixed cells at high density with molten top agar, then overlaid this mixture on a hard agar substratum. Such an approach yielded highly-reproducible, immobilized, soft agar-embedded “lawns” of cells.

### 3.1. Tolerance to Antibiotics

Depending on the mechanism of function, different antibiotics (Figure S1) exert their effects in different subcellular zones; however, they must all first encounter the bacterial cell surface prior to any subsequent effected actions. The polymyxins directly target the cell-surface of Gram-negative bacteria; therein, a polycationic peptide ring interacts with lipopolysaccharide (LPS) in the outer leaflet of the OM and displaces the divalent cations that bridge individual LPS molecules, while a fatty acid tail is able to insert into the hydrophobic layer of the OM, together causing lethal OM permeability defects. Drugs such as vancomycin and ampicillin act in the periplasm as they impede peptidoglycan (PG) cross-linking, the former via binding to D-Ala-D-Ala growing-peptide termini, and the latter via inactivation of penicillin-binding proteins exposed at the periplasmic leaflet of the IM. Finally, antibiotics such as ciprofloxacin and chloramphenicol must gain access to the cytoplasm where they impede the synthesis of DNA (via binding to topoisomerases II and IV) and protein (via binding the 50S ribosomal subunit), respectively [30].

#### 3.1.1. Tolerance of Cells That Produce a Thicker EPS Glycocalyx

**Figure 2:**
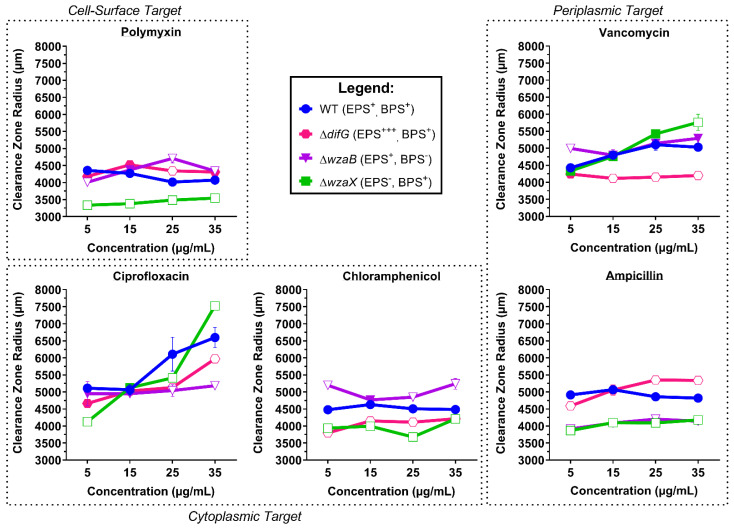
Radial measurements of clearance zones for antibiotic-treated *M. xanthus* polysaccharide-mutant strains. Data points represent means from three biological replicates (±SEM). Open and closed data points for mutant strains represent mean values with and without statistically significant differences (respectively) relative to the WT value at a given concentration, as determined via two-way ANOVA with Dunnett’s multiple comparisons test (α ≤ 0.05).

While tolerance to Polymyxin B, ampicillin, and ciprofloxacin were unchanged relative to WT, an over-abundance of cell-surface EPS was found to render Δ*difG* cells less susceptible to killing by vancomycin and inhibition by chloramphenicol (Figure 2).

Given the large size of vancomycin (Figure S1), the higher tolerance of Δ*difG* cells may be due to a steric impediment preventing surface access for this glycopeptide drug. This would also explain the reduced chloramphenicol susceptibility as this drug is particularly hydrophobic. Considering Δ*difG* cells were previously found [8] to be relatively more hydrophobic than WT (Figure 1), Δ*difG* cells could be hypothesized to be even more susceptible than WT cells to inhibition by chloramphenicol. As the opposite is true, this would bolster the notion of a structural barrier preventing cell-surface access of this drug.

#### 3.1.2. Tolerance of Cells That Produce a Non-Disrupted EPS Glycocalyx

Despite a lack of BPS production in Δ*wzaB*, cells of this strain were previously shown to still elaborate WT amounts of cell-surface EPS [7]. However, in the absence of BPS secretion, the EPS glycocalyx manifested a more contiguous, non-disrupted physical state [8], leading to higher-than-WT levels of Trypan Blue dye binding [7], indicating that a non-disrupted EPS layer may be able to better retain certain molecules. From the antibiotic-susceptibility data shown herein (Figure 2), Δ*wzaB* cells demonstrated WT-like tolerance of Polymyxin B and vancomycin. However, Δ*wzaB* cells were significantly less susceptible to killing by ampicillin or ciprofloxacin, suggesting that the envelope properties of Δ*wzaB* cells were an impediment to the uptake and/or action of the two drugs. Intriguingly, Δ*wzaB* cells with a non-disrupted EPS glycocalyx were found to be less tolerant of chloramphenicol and were thus inhibited more than WT; this is of note as none of the other strains tested displayed such a profile. Given the hydrophobic character of chloramphenicol, and the previous observation that WT cells have a higher relative hydrophobicity than Δ*wzaB* cells [8], it would have been expected that WT cells were better inhibited than those of Δ*wzaB*; however, as this was not so, these data point towards EPS-layer electrostatic character not playing a major role in chloramphenicol access to the cell.

#### 3.1.3. Tolerance of Cells That Do Not Produce an EPS Glycocalyx

**Figure A2:**
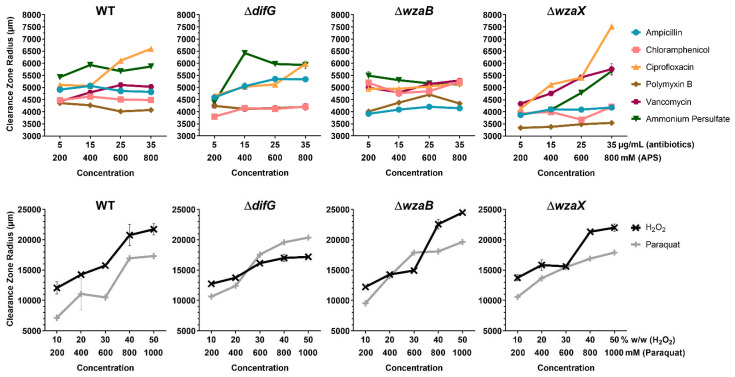
Strain-specific radial measurements of clearance zones for *M. xanthus* polysaccharide mutants strains treated with antibiotics and oxidative stress-inducing agents. All points represent means from three biological replicates (±SEM). Data comparing each compound amongst the various strains tested is presented in Figure 2 and Figure 3.

As seen from the different antibiotic susceptibility profiles for Δ*wzaX* (relative to WT), the mere absence an EPS glycocalyx can have different effects depending on the drug in question (Figure A2). Compared to both WT and Δ*wzaB*, EPS^−^ Δ*wzaX* cells displayed no differences in susceptibility to vancomycin. However, Δ*wzaX* cells treated with Polymyxin B, ampicillin, chloramphenicol, or ciprofloxacin all demonstrated less drug susceptibility than WT cells, indicating that the overall presence of an EPS glycocalyx confers increased tolerance. Of note, for ciprofloxacin treatment, EPS^−^ Δ*wzaX* cells demonstrated the lowest susceptibility to killing at the lowest drug concentration tested, whereas at the highest drug concentration, these cells became the most susceptible to killing (Figure 2). This could indicate that the EPS glycocalyx has a retention capacity for molecules, as well as a straightforward barrier function; this would explain lower ciprofloxacin sensitivity at low concentrations compared to all other strains tested, whereas at higher concentrations, Δ*wzaX* cells become the most susceptible among all strains tested. In essence, EPS^−^ cells are less able to buffer different conditions in the extracellular milieu.

### 3.2. Tolerance to Reactive Oxygen Species

Similar to antibiotics, different agents that effect damage via oxidative stress can exert this stress in different subcellular zones. At the cell surface, hydrogen peroxide (H_2_O_2_) has been shown to increase membrane permeability and oxidize proteins [31]. Similarly, ammonium persulfate (APS) is able to oxidize bacterial matrix polysaccharides [32]. H_2_O_2_ can also pass through the cell wall and interact with iron (Fe^2+^) via a Fenton reaction, producing reactive oxygen species (ROS) in the form of hydroxyl radicals (HO•) that can provoke damage to DNA and other intracellular contents [33]. Redox-cycling drugs such as paraquat can enter the bacterial cell, strip electrons from redox enzymes, and transfer them to oxygen to form ROS such as superoxide (O_2_^−^) [34]. Regardless of subcellular localization, the presence of ROS is toxic to bacterial cells.

#### 3.2.1. Tolerance of Cells That Produce a Thicker EPS Glycocalyx 

**Figure 3:**
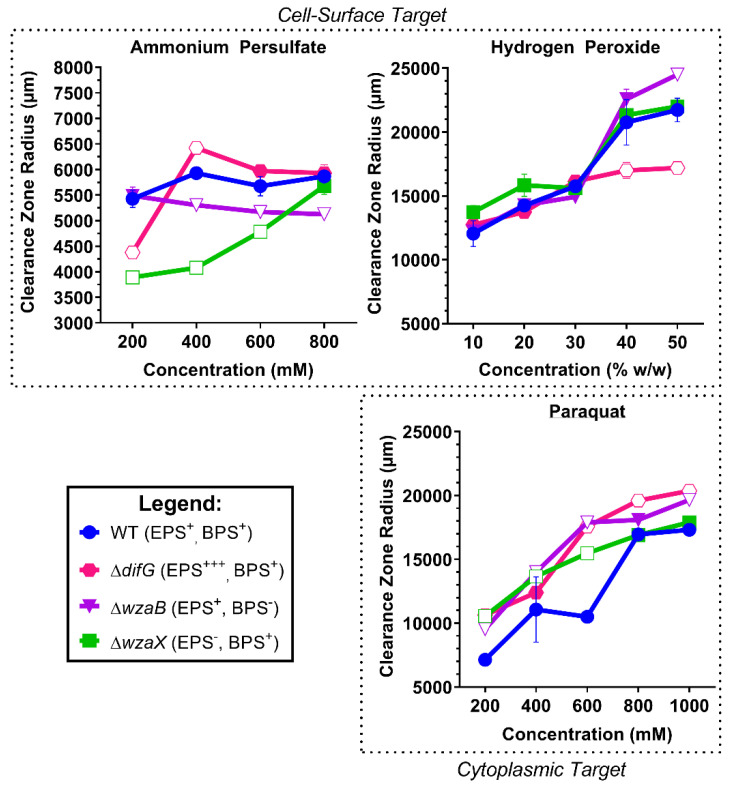
Radial measurements of clearance zones for *M. xanthus* polysaccharide-mutant strains treated with oxidative stress-inducing agents. Data points represent means from three biological replicates (±SEM). Open and closed points for mutant strains represent means with and without statistically significant differences (respectively) relative to the WT value at a given concentration, as determined via two-way ANOVA with Dunnett’s multiple comparisons test (α ≤ 0.05).

Despite an overabundance of EPS on the surface, Δ*difG* cells were as susceptible as WT cells to killing by APS. Tolerance for both of these strains to paraquat was dose-dependent, albeit with Δ*difG* cells displaying slightly higher susceptibility throughout. Intriguingly though, Δ*difG* was the only strain that displayed higher tolerance than WT when exposed to H_2_O_2_, suggesting the thicker layer of cell-surface EPS may have contributed to reduced sensitivity of this strain to killing by ROS outside the cell (Figure 3).

#### 3.2.2. Tolerance of Cells That Produce a Non-Disrupted EPS Glycocalyx 

Cells that produce a non-disrupted EPS glycocalyx (Δ*wzaB*) were (i) more tolerant to oxidation by APS compared to WT (or Δ*difG*), yet (ii) more susceptible to killing at higher concentrations of H_2_O_2_ and paraquat. Killing by paraquat was highly reflective of the overall dose-dependent effect for all strains (Figure 3). Overall, similar to antibiotics (Figure 2), EPS glycocalyx integrity impacts tolerance to killing by oxidative-stress agents.

#### 3.2.3. Tolerance of Cells That Do Not Produce an EPS Glycocalyx 

While cells that do not produce an EPS glycocalyx (Δ*wzaX*) displayed WT-like H_2_O_2_ and paraquat susceptibility, tolerance to APS was dose-dependent, with EPS^−^ cells the most tolerant to APS (of all strains tested) at lower APS concentrations (Figure 3). In this manner, a lack of cell-surface EPS was beneficial for survival in the presence of APS.

## 4. Discussion

The presence of a glycocalyx between the OM and the extracellular milieu is naturally a protective physical barrier which prevents immediate contact of toxic compounds with the surface of the cell. Herein, the presence of excess cell-surface EPS material (Δ*difG* cells) was largely of benefit to *M. xanthus*, as it conferred WT-level-or-higher tolerance to Polymyxin B, ampicillin, ciprofloxacin, chloramphenicol, vancomycin, APS, and H_2_O_2_. The only compound to which Δ*difG* cells were consistently more susceptible than WT cells was paraquat. This could be explained by heightened retention of paraquat in this mutant background via a sponge-like effect. Given that paraquat is continually regenerated once inside cells, this would be consistent with the higher-than-WT killing in Δ*difG* cells.

Previously, cells with a non-disrupted EPS glycocalyx (Δ*wzaB*) were found to retain more Trypan Blue dye than WT, despite having the same amount of cell-surface EPS [7], suggesting that the EPS layer in Δ*wzaB* cells may manifest better retention properties for certain compounds. Incidentally, Δ*wzaB* cells were more susceptible to chloramphenicol, H_2_O_2_, and paraquat than WT, the only mutant tested to display such a profile. Conversely, the Δ*wzaB* strain displayed a higher tolerance to ampicillin, ciprofloxacin, and APS compared to WT, consistent with the envelope properties in this mutant being detrimental to the action of either drug. Thus, the integrity of the EPS glycocalyx is an important mediator of tolerance to antibiotics and oxidative stress-inducing compounds.

This investigation sheds light on the importance of disrupting cell-surface EPS layers, an act which could be envisioned to improve outcomes in host infection settings. The development of EPS-disrupting compounds could thus improve patient outcomes when used in conjunction with a suitable antibiotic. EPS also protects bacteria against oxidative stress in the soil, such as for plant microsymbionts [35]. Based on our findings, we posit that the integrity of the glycocalyx in such environmental settings is also of importance, particularly given that many toxic compounds for bacteria (e.g., antibiotics, ROS, etc.) can be found in the soil [36,37]. This work also opens the door for improvements in engineering strategies for plant and animal microbiomes to improve health [38] through the consideration of the presence and state of HMW polysaccharides in these systems.

## Figures and Tables

**Table 1:** *Myxococcus xanthus* strains used in this study.

Strain Code	Genotype/Description	Reference
DZ2	*M. xanthus* (wild type)	[28]
TM469	Δ*wzaX* (Δ*mxan_7417/epsY/MXDZ_RS0233025*)	[29]
TM529	Δ*wzaB* (Δ*mxan_1915/MXDZ_RS0224835*)	[29]
EM451	Δ*difG* (Δ*mxan_6691/MXDZ_RS0216165*)	[8]

## Data Availability

The data that support the findings of this study are available from the corresponding author upon reasonable request.

## Supplementary Materials

The following supporting information can be downloaded at: https://www.mdpi.com/article/10.3390/biom12040571/s1, Figure S1: Chemical structures of the antibiotics used in this study.

## Appendix A

Cells of *M. xanthus* can be evenly distributed and immobilized within soft agar cast atop a hard-agar matrix, allowing for disc-diffusion testing of various compounds.

## Appendix B

When tolerances to different compounds were compared for each strain, H_2_O_2_ and paraquat displayed dose-dependent effects, the severity of which were strain-dependent (Figure A2). A similar effect was observed for most antibiotics in Δ*wzaX* (EPS^−^), whereas those with an EPS glycocalyx were better buffered against each antibiotic (Figure A2).
