# Supplementary figures and images for: Bacterial Glycocalyx Integrity Impacts Tolerance of Myxococcus xanthus to Antibiotics and Oxidative-Stress Agents
Source: Biomolecules. 2022 Apr 12;12(4):571. doi: 10.3390/biom12040571 (PMC9029694; doi:10.3390/biom12040571)

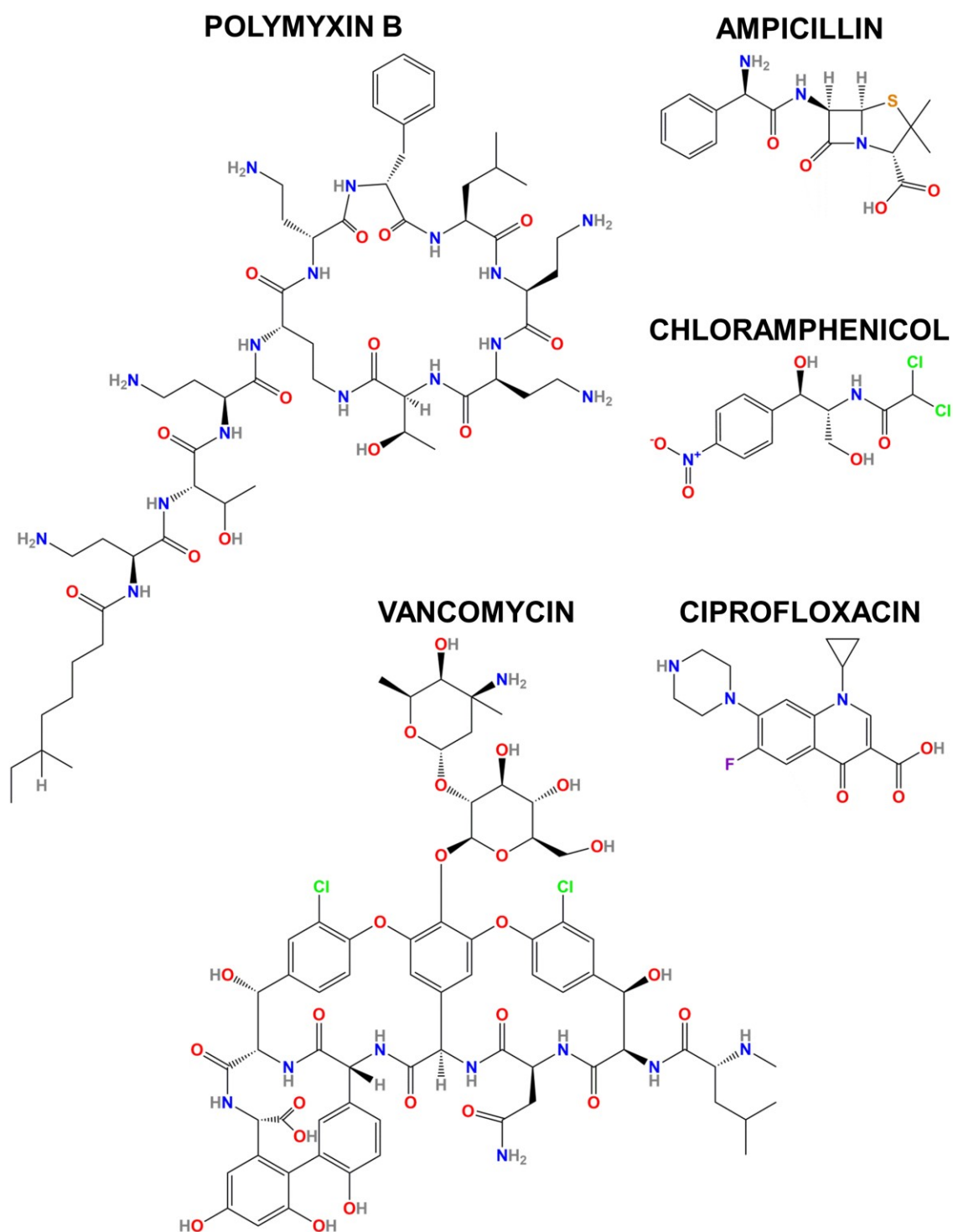

**Figure S1.** Chemical structures of the antibiotics used in this study.

Supplement: Supplementary file 1 [file biomolecules-12-00571-s001.zip › biomolecules-1662465-supplementary.pdf]
